# ASO Author Reflections: Early Recurrence After Esophageal Cancer Resection Cannot be Predicted Preoperatively: A Call for More Reliable Biomarkers

**DOI:** 10.1245/s10434-024-16466-4

**Published:** 2024-11-18

**Authors:** Ingmar F. Rompen, Adrian T. Billeter, Nerma Crnovrsanin, Leila Sisic, Kerstin J. Neuschütz, Julian Musa, Martin Bolli, Lana Fourie, Marko Kraljevic, Mohammed Al-Saeedi, Henrik Nienhüser, Beat P. Müller-Stich

**Affiliations:** 1 Department of General, Visceral and Transplantation Surgery, Heidelberg University Hospital, Heidelberg, Germany; 2 Department of Surgery, Clarunis-University Digestive Health Care Center, St. Clara Hospital and University Hospital Basel, Basel, Switzerland

## Past

Surgical resection combined with perioperative chemotherapy is the mainstay of treatment for patients with radiologically localized esophageal cancer. However, approximately one in two patients will experience recurrence, predominantly at a distant site.^1,2^ Hereby, if an early recurrence is diagnosed, the benefit of local treatment is often questioned by both the patient and the treating physician. Furthermore, if at high risk, a patient may benefit from closer follow-up to treat aggressive tumor biology in form of early recurrence early on.^1^ To date, three challenges need to be addressed: (a) Is there a statistically sound cutoff for early versus late recurrence? (b) Can we predict early recurrence? and (c) Can we influence the occurrence of early recurrence with treatment modalities?

## Present

In the present analysis, 1.5 years after resection was the optimal statistical cutoff for separating early from late recurrence.^3^ Preoperatively only clinically nodal positive disease was associated with early recurrence with a positive predictive value of 95% but a poor negative predictive value of 18%. Postoperatively, remaining advanced disease (ypT 3–4, ypN1–3, and minor pathological treatment response) was independently associated with early recurrence. Except from providing adjuvant therapy, treatment modalities such as type of neoadjuvant treatment or surgical approach have no clinically relevant influence on early recurrence and recurrence overall. This is in line with Donlon et al. comparing CROSS versus FLOT, who found no clear evidence for better local control via radiotherapy or better systemic control via aggressive systemic treatment with FLOT.^4^ Although recurrence rates and sites have not yet been published, these findings are challenged by the recently presented ESOPEC trial, suggesting improved survival with FLOT compared with CROSS.

## Future

These results demonstrate that we do not yet have reliable preoperative biomarkers to predict early recurrence. Therefore, decisions against surgical resection should not be based on the presence of advanced disease and presumed unfavorable tumor biology. Postoperatively, those with advanced residual disease may benefit from closer follow-up compared with those who are not at high risk. In the future, more reliable biomarkers are needed to guide the treatment of patients with esophageal cancer. The introduction of PD-1 directed immunotherapy may have the potential to provide the long-awaited improvement in systemic disease control.^5^
